# Assessment of Self-reported Prognostic Expectations of People Undergoing Dialysis: United States Renal Data System Study of Treatment Preferences (USTATE)

**DOI:** 10.1001/jamainternmed.2019.2879

**Published:** 2019-07-08

**Authors:** Ann M. O’Hare, Manjula Kurella Tamura, Danielle C. Lavallee, Elizabeth K. Vig, Janelle S. Taylor, Yoshio N. Hall, Ronit Katz, J. Randall Curtis, Ruth A. Engelberg

**Affiliations:** 1 Department of Medicine, University of Washington, Seattle; 2 Kidney Research Institute, University of Washington, Seattle; 3 VA Puget Sound Health Care System, Seattle, Washington; 4 Stanford University Medical Center, Palo Alto, California; 5 Division of Nephrology, Geriatric Research, Education and Clinical Center, VA Palo Alto Health Care System, Palo Alto, California; 6 Department of Surgery, University of Washington, Seattle; 7 Department of Anthropology, University of Washington, Seattle; 8 Cambia Palliative Care Center of Excellence, University of Washington, Seattle

## Abstract

**Importance:**

Prognostic understanding can shape patients’ treatment goals and preferences. Patients undergoing dialysis in the United States have limited life expectancy and may receive end-of-life care directed at life extension. Little is known about their prognostic expectations.

**Objective:**

To understand the prognostic expectations of patients undergoing dialysis and how these relate to care planning, goals, and preferences.

**Design, Setting, and Participants:**

Cross-sectional survey study of 996 eligible patients treated with regular dialysis at 31 nonprofit dialysis facilities in 2 metropolitan areas (Seattle, Washington, and Nashville, Tennessee) between April 2015 and October 2018. After a pilot phase, 1434 eligible patients were invited to participate (response rate, 69.5%). To provide a context for interpreting survey participants’ prognostic estimates, United States Renal Data System standard analysis files were used to construct a comparison cohort of 307 602 patients undergoing in-center hemodialysis on January 1, 2006, and followed for death through July 31, 2017. Final analyses for this study were conducted between November 2018 and March 2019.

**Exposures:**

Responses to the question “How long would you guess people your age with similar health conditions usually live?” (<5 years, 5-10 years, >10 years, or not sure).

**Main Outcomes and Measures:**

Self-reported (1) documentation of a surrogate decision-maker, (2) documentation of treatment preferences, (3) values around life prolongation, (4) preferences for receipt of cardiopulmonary resuscitation and mechanical ventilation, and (5) desired place of death.

**Results:**

Of the 996 survey respondents, the mean (SD) age was 62.7 (13.9) years, and 438 (44.0%) were women. Overall, 112 (11.2%) survey respondents selected a prognosis of fewer than 5 years, 150 (15.1%) respondents selected 5 to 10 years, 330 (33.1%) respondents selected more than 10 years, and 404 (40.6%) were not sure. By comparison, 185 427 (60.3%) prevalent US in-center patients undergoing hemodialysis died within 5 years, 58 437 (19.0%) died within 5 to 10 years, and 63 738 (20.7%) lived more than 10 years. In analyses adjusted for participant characteristics, survey respondents with a prognostic expectation of more than 10 years (vs <5 years) were less likely to report documentation of a surrogate decision-maker (adjusted odds ratio [aOR], 0.6; 95% CI, 0.4-0.9) and treatment preferences (aOR, 0.4; 95% CI, 0.2-0.6) and to value comfort over life extension (aOR, 0.1; 95% CI, 0.04-0.3), and were more likely to want cardiopulmonary resuscitation (aOR, 5.3; 95% CI, 3.2-8.7) and mechanical ventilation (aOR, 2.2; 95% CI, 1.2-3.7). The respondents who reported that they were not sure about prognosis had similar associations.

**Conclusions and Relevance:**

Uncertain and overly optimistic prognostic expectations may limit the benefit of advance care planning and contribute to high-intensity end-of-life care in patients undergoing dialysis.

## Key Points

**Question:**

What are the prognostic expectations of people undergoing dialysis, and how do these relate to their treatment goals and preferences?

**Findings:**

In this cross-sectional survey study of 996 patients receiving maintenance dialysis at nonprofit facilities in 2 US metropolitan areas, most of the respondents were either uncertain about prognosis or had a prognostic expectation of more than 10 years. In adjusted analyses, these groups were less likely than those with a prognostic expectation of fewer than 5 years to report having documented their treatment preferences and to value comfort over life extension, and more likely to want cardiopulmonary resuscitation and mechanical ventilation.

**Meaning:**

Prognostic uncertainty and overly optimistic prognostic expectations among people undergoing dialysis may limit the benefit of advance care planning and contribute to intensive patterns of end-of-life care.

## Teaser

This cross-sectional survey study assesses the self-reported prognostic expectations of patients undergoing dialysis and how these expectations relate to care planning, goals, and preferences.

## Introduction

Patients’ understanding of their prognosis and expected course of illness can profoundly shape their treatment goals and life choices.^1^ Such understanding is essential to the delivery of patient-centered care, shared decision-making, and advance care planning.^2,3,4^ Available evidence in people with cancer and older adults suggests that clinicians rarely share prognostic information^5,6,7,8,9^ and that patients often have overly optimistic expectations about the future.^1,5,6,7,8,9^

People with advanced kidney disease who undergo maintenance dialysis have limited life expectancy. Compared with other seriously ill populations, rates of advance care planning among members of this population are lower^10,11^ and patterns of end-of-life care are more focused on life prolongation.^12,13,14,15,16^ Although people with advanced kidney disease report wanting to learn about their prognosis and future course of illness,^17,18,19,20,21^ nephrologists rarely share prognostic information with their patients.^22,23,24,25^

Prior studies suggest that people with advanced kidney disease tend to have overly optimistic expectations about the future.^21,24,26,27^ However, these earlier studies were limited because they did not frame prognosis in terms of life expectancy, offer patients an opportunity to indicate if they were uncertain about their prognosis, or provide a detailed characterization of how prognostic expectations relate to other domains of end-of-life care.

In this study, we report on a survey that elicited the prognostic expectations of people undergoing dialysis framed in terms of life expectancy and examine how these expectations relate to engagement in advance care planning, treatment preferences, and values around life prolongation. To provide a context for interpreting the survey results, we compared participants’ prognostic expectations with actuarial survival in the US dialysis population.

## Methods

### Recruitment of Survey Participants

Study participants were identified from 31 nonprofit dialysis facilities in 2 US metropolitan areas (Seattle, Washington, and Nashville, Tennessee). Patients receiving dialysis in these facilities were eligible to participate in the study if they met the following criteria: (1) 21 years or older; (2) sufficiently fluent to communicate in English; and (3) cognitively able to provide informed consent. Study staff consulted with dialysis facility charge nurses to identify patients who met these eligibility criteria. Staff then recruited a pragmatic consecutive sample of eligible patients receiving dialysis at each facility at the time of survey administration. Enrolled patients completed a survey that included questions about prognostic expectations, readiness to engage in advance care planning, values around life prolongation, treatment preferences, and preferred place of death (Supplement).

Between April 2015 and October 2018, we approached 1595 patients to participate in the study (eFigure in the Supplement). During the first 8 months of recruitment, 161 eligible patients were invited to participate in the pilot phase of the study, and 146 patients completed pilot versions of the survey (90.6% response rate). Following the pilot phase, a further 1434 eligible patients were invited to participate, and 1009 patients completed the final version of the survey. We excluded 13 of these respondents because they did not record their name and/or date of birth on the paper survey or consent form (n = 9) or did not respond to the prognosis question (n = 4), yielding an analytic cohort of 996 patients (69.5% response rate). Of these, 991 (99.5%) were receiving in-center hemodialysis; the other 5 (0.5%) were receiving peritoneal dialysis. This study was approved by the institutional review board at the University of Washington in Seattle, and participants provided written informed consent.

### Survey Design and Administration

The survey was designed by 5 of the authors (A.M.O., R.A.E., D.C.L., M.K.T., and E.K.V.). Survey items related to treatment preferences, values, and preferred place of death were taken or adapted from existing instruments.^24,26,28,29^ During the first 8 months of administration, the survey was iteratively refined to improve clarity and reduce length based on feedback from the first 146 patients enrolled in the study, staff members administering the survey, 2 patients with end-stage renal disease (ESRD) who were not enrolled in the study, the widow of a patient with ESRD, and a long-time patient advocate. During pilot testing, we asked patients to list any questions they found upsetting or difficult to understand and used this information to refine the survey. Participants could choose to have study coordinators record their survey responses during their dialysis session or they could complete the survey themselves and return it in person or by mail.

### Primary Exposure

Responses to the following question served as the primary exposure: “How long would you guess people your age with similar health conditions usually live?” Possible answers included “less than 6 months,” “6 to 12 months,” “1 to 2 years,” “2 to 5 years,” “5 to 10 years,” “more than 10 years,” and “I’m not sure.” For responses that straddled categories, participants were instructed to select the higher category. Because few respondents expected to live fewer than 2 years (n = 41), response categories were analyzed as fewer than 5 years, 5 to 10 years, more than 10 years, or not sure.

### Covariates

Self-reported participant characteristics included age, sex, race (white, black, Asian, American Indian or Alaskan Native, Native Hawaiian or other Pacific Islander, other, or missing), ethnicity (Hispanic or non-Hispanic), health status (excellent, very good, good, fair, or poor), time undergoing dialysis, and highest level of educational attainment (eighth grade or less; some high school; completed high school or equivalent; some college, community college, or trade school; graduated from college, community college, or trade school; or postgraduate training). We assessed self-reported spirituality based on responses to the following statement: “My religious or spiritual beliefs are really what lie behind my whole approach to life.” Possible answers included “definitely true,” “tends to be true,” “tends not to be true,” and “definitely not true.”

### Outcomes

We examined the following outcomes: (1) self-reported documentation of a surrogate decision-maker (“I have signed official papers” vs “I have not thought about this,” “I have thought about this but not decided who,” “I know who this would be but have not asked him/her,” or “I have asked someone but have not signed official papers”), (2) self-reported documentation of treatment preferences (“I have signed official papers” vs “I have not thought about this,” “I have thought about this but have not talked to anyone about it,” “I have talked about this with a friend or family member but have not signed official papers,” or “I have talked about this with a doctor or other health care provider but have not signed official papers”), (3) values around life prolongation (participants were asked about their preferred plan of care if they were to become “very sick in the future” with possible responses including “extending life, even if that means having more pain and discomfort,” “relieving pain and discomfort as much as possible, even if that means not living as long,” or “I’m not sure which I would choose”), (4) preferences for receipt of cardiopulmonary resuscitation (CPR) and mechanical ventilation (participants were asked if they would want these interventions if they “had to decide right now” with possible responses including “definitely yes,” vs “probably yes,” “probably not,” or “definitely not,”), and (5) desired place of death (home or home of a relative or friend vs hospital, nursing home, or other).

### Survival Among Prevalent US Patients Undergoing Dialysis

To provide a context for interpreting survey participants’ prognostic estimates, we used United States Renal Data System (USRDS) standard analysis files to construct a comparison cohort of 307 602 patients 21 years or older who were receiving in-center hemodialysis on January 1, 2006 (to allow for at least 10 years of follow-up). Among members of this cohort, we described actuarial survival through July 31, 2017; we also examined survival among the subset of cohort members who received a transplant during follow-up. Treatment modality was ascertained from the USRDS Treatment History File, and age, sex, race, ethnicity, time since onset of ESRD, date of first transplant, and date of death were ascertained from the USRDS Patients File.

### Statistical Analyses

The characteristics of survey participants were described both for the overall cohort and after stratification by patients’ prognostic expectations (<5 years, 5-10 years, >10 years, and not sure). We used multinomial regression to examine the associations between prognostic expectations and the following self-reported participant characteristics: age, sex, race, ethnicity, health status, time since ESRD onset, highest educational level, and spirituality. We used logistic and multinomial regression adjusted for these same self-reported characteristics to measure the associations of prognostic expectations with study outcomes. All analyses were conducted using Stata, version 13.1 (StataCorp).

## Results

### Characteristics and Prognostic Expectations of Survey Respondents

Survey respondents had a mean (SD) age of 62.7 (13.9) years, 438 (44.0%) were women, 565 (56.7%) were white, 268 (26.9%) were black, and 64 (6.4%) were Hispanic (Table 1). At the time of survey completion, most participants reported being in excellent, very good, or good health (n = 576; 57.8%), had been treated with dialysis for fewer than 5 years (n = 757; 76.0%), and had some college or postgraduate education (n = 531; 53.3%). The majority (n = 708; 71.1%) indicated that the statement “my religious or spiritual beliefs are really what lie behind my whole approach to life” was “definitely true” or “tends to be true.”

**Table 1.** Characteristics of USTATE Participants Stratified by Prognostic Expectations^a^

Characteristics	Cohort Patients (n = 996), No. (%)	Prognostic Expectations, No. (%)				*P* Value^b^
		<5 y (n = 112)	5-10 y (n = 150)	>10 y (n = 330)	Uncertain (n = 404)	
Age group, y						<.001
<45	113 (11.4)	1 (0.9)	11 (7.3)	63 (19.1)	38 (9.4)	
45-59	289 (29.0)	24 (21.4)	29 (19.3)	115 (34.9)	121 (30.0)	
60-74	408 (41.0)	51 (45.5)	74 (49.3)	127 (38.5)	156 (38.6)	
≥75	186 (18.7)	36 (32.1)	36 (24.0)	25 (7.6)	89 (22.0)	
Female sex	438 (44.0)	46 (41.1)	73 (48.7)	121 (36.7)	198 (49.0)	.005
Race						.02
White	565 (56.7)	83 (74.1)	97 (64.7)	181 (54.9)	204 (50.5)	
Black	268 (26.9)	15 (13.4)	36 (24.0)	95 (28.8)	122 (30.2)	
Asian	83 (8.3)	8 (7.1)	8 (5.3)	26 (7.9)	41 (10.2)	
American Indian or Alaskan Native	16 (1.6)	2 (1.8)	1 (0.7)	7 (2.1)	6 (1.5)	
Native Hawaiian or other Pacific Islander	31 (3.1)	2 (1.8)	4 (2.7)	9 (2.7)	16 (4.0)	
Other or missing	33 (3.3)	2 (1.8)	4 (2.7)	12 (3.6)	15 (3.7)	
Ethnicity						.07
Hispanic	64 (6.4)	5 (4.5)	6 (4.0)	28 (8.5)	25 (6.2)	
Missing	15 (1.5)	0	1 (0.7)	3 (0.9)	11 (2.7)	
Self-reported health status						<.001
Excellent	33 (3.3)	4 (3.6)	3 (2.0)	10 (3.0)	16 (4.0)	
Very good	163 (16.4)	9 (8.0)	24 (16.0)	72 (21.8)	58 (14.4)	
Good	380 (38.2)	28 (25.0)	54 (36.0)	147 (44.6)	151 (37.4)	
Fair	310 (31.1)	43 (38.4)	49 (32.7)	78 (23.6)	140 (34.7)	
Poor	108 (10.8)	28 (25.0)	20 (13.3)	23 (7.0)	37 (9.2)	
Missing	2 (0.2)	0	0	0	2 (0.5)	
Time undergoing dialysis						.95
<6 mo	133 (13.4)	14 (12.5)	23 (15.3)	45 (13.6)	51 (12.6)	
6-12 mo	145 (14.6)	17 (15.2)	26 (17.3)	41 (12.4)	61 (15.1)	
1-2 y	216 (21.7)	27 (24.1)	35 (23.3)	74 (22.4)	80 (19.8)	
2-5 y	263 (26.4)	28 (25.0)	36 (24.0)	89 (27.0)	110 (27.2)	
5-10 y	168 (16.9)	19 (17.0)	22 (14.7)	53 (16.1)	74 (18.3)	
>10 y	68 (6.8)	7 (6.3)	7 (4.7)	27 (8.2)	27 (6.7)	
Missing	3 (0.3)	0	1 (0.7)	1 (0.3)	1 (0.3)	
Highest educational level						.001
≤8th grade	31 (3.1)	1 (0.9)	3 (2.0)	4 (1.2)	23 (5.7)	
Some high school	96 (9.6)	9 (8.0)	9 (6.0)	26 (7.9)	52 (12.9)	
Graduated high school/GED	331 (33.2)	38 (33.9)	45 (30.0)	98 (29.7)	150 (37.1)	
Some college	169 (17.0)	15 (13.4)	22 (14.7)	69 (20.9)	63 (15.6)	
Graduated from college	307 (30.8)	37 (33.0)	56 (37.3)	113 (34.2)	101 (25.0)	
Postgraduate education	55 (5.5)	11 (9.8)	14 (9.3)	18 (5.5)	12 (3.0)	
Missing or other	7 (0.7)	1 (0.9)	1 (0.7)	2 (0.6)	3 (0.8)	
Religious and spiritual beliefs important						.27
Definitely true	466 (46.8)	38 (33.9)	77 (51.3)	162 (49.1)	189 (46.8)	
Tends to be true	242 (24.3)	33 (29.5)	32 (21.3)	78 (24.6)	99 (24.5)	
Tends not to be true	140 (14.1)	21 (18.8)	25 (16.7)	40 (12.1)	54 (13.4)	
Definitely not true	138 (13.9)	19 (17.0)	15 (10.0)	48 (14.6)	56 (13.9)	
Missing	10 (1.0)	1 (0.9)	1 (0.7)	2 (0.6)	6 (1.5)	

Abbreviations: GED, General Educational Development test; USTATE, United States Renal Data System Study of Treatment Preferences.

^a^ Columns may not sum to 100% owing to rounding.

^b^ *P* values calculated using a χ^2^ test.

**Figure 1.**
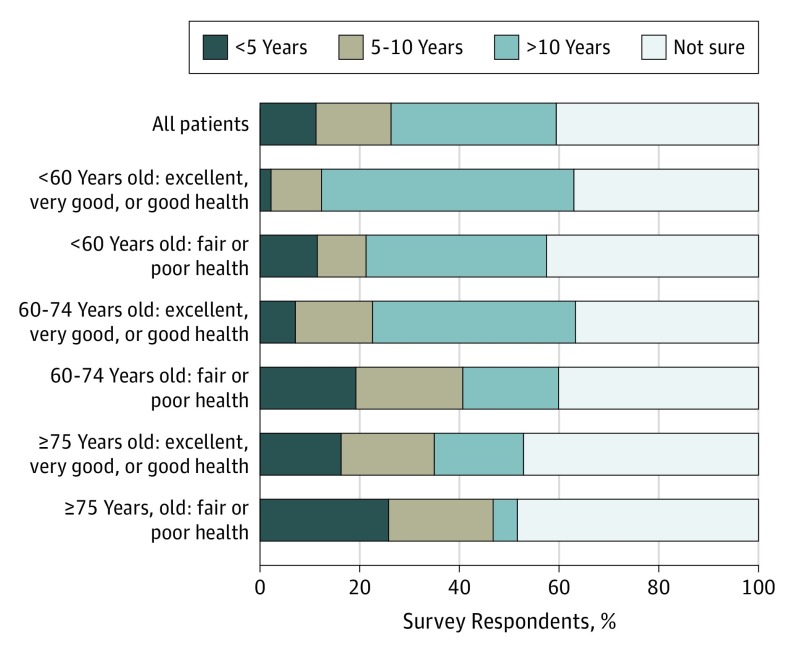
Prognostic Expectations of Survey Participants Overall and Stratified by Age and Self-reported Health Status

When participants were asked to guess how long they thought people of their age with similar health conditions usually live, 112 (11.2%) responded fewer than 5 years, 150 (15.1%) responded 5 to 10 years, 330 (33.1%) responded more than 10 years, and 404 (40.6%) were not sure (Figure 1). Even among the 62 participants 75 years or older with fair or poor self-reported health, 16 (25.8%) responded fewer than 5 years, 13 (21.0%) responded 5 to 10 years, 3 (4.8%) responded more than 10 years, and 30 (48.4%) were not sure.

### Characteristics and Survival of Prevalent US Patients Undergoing In-Center Hemodialysis

**Figure 2.**
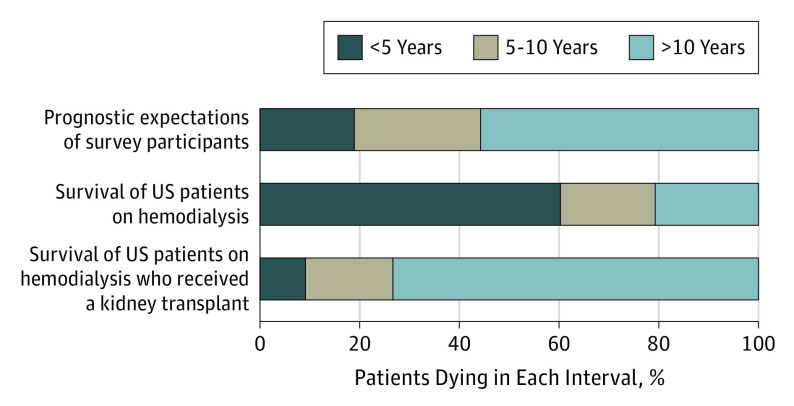
Prognostic Expectations of Survey Participants and Actuarial Survival Among Prevalent US In-Center Patients Undergoing Hemodialysis To facilitate comparisons across groups, survey participants who were not sure of their prognosis are excluded.

The mean (SD) age of prevalent in-center US patients undergoing hemodialysis (n = 307 602) on January 1, 2006, was 62.0 (15.0) years, 45.3% were women, 55.1% were white, 38.0% were black, and 15.7% were Hispanic (eTable 1 in the Supplement). A majority (60.3%) of these patients died within 5 years of the index date, 19.0% died within 5 to 10 years, and 20.7% lived more than 10 years (Figure 2).

A subset of 33 713 (11.0%) patients in the cohort went on to receive a kidney transplant during follow-up. The mean (SD) age of these patients on January 1, 2006, was 49.5 (12.6) years, 36.7% were women, 51.7% were white, 39.7% were black, and 21.0% were Hispanic (eTable 1 in the Supplement). Of these patients, 9.1% died within 5 years, 17.6% died within 5 to 10 years, and 73.3% lived more than 10 years (Figure 2).

### Advance Care Planning, Treatment Preferences, Values, and Preferred Place of Death

Overall, 483 (48.5%) survey participants reported having documented a surrogate decision-maker, and 376 (37.8%) reported having documented their treatment preferences (Table 2). Most participants reported that they would “definitely” or “probably” want CPR (n = 837; 84.0%) and mechanical ventilation (n = 631; 63.3%). Almost half (n = 481; 48.3%) said that they would prioritize relieving pain and discomfort over extending life, and almost one-third (n = 319; 32.0%) were uncertain. A majority (n = 574; 57.6%) preferred to die at home or at the home of a relative or friend.

**Table 2.** Engagement in Advance Care Planning, Values, Treatment Preferences, and Preferred Place of Death Among USTATE Participants With Differing Prognostic Expectations^a^

Questions and Responses	Cohort Patients (n = 996), No. (%)	Prognostic Expectations, No. (%)				*P* Value^b^
		<5 y (n = 112)	5-10 y (n = 150)	>10 y (n = 330)	Uncertain (n = 404)	
**Do you have a person who could make medical decisions for you if you were to become very sick and were unable to speak for yourself?**						
I have not thought about this	57 (5.7)	7 (6.3)	5 (3.3)	21 (6.4)	24 (5.9)	.002
I have thought about this but have not decided who	60 (6.0)	4 (3.6)	8 (5.3)	25 (7.6)	23 (5.7)	
I know who this would be but have not asked him/her	76 (7.6)	4 (3.6)	5 (3.3)	34 (10.3)	33 (8.2)	
I have asked someone but have not signed official papers	304 (30.5)	27 (24.1)	41 (27.3)	114 (34.6)	122 (30.2)	
I have signed official papers	483 (48.5)	70 (62.5)	91 (60.7)	130 (39.4)	192 (47.5)	
Missing	16 (1.6)	0	0	6 (1.8)	10 (2.5)	
**Have you thought about the kinds of treatments that you would want or not want if you were to become very sick and were unable to speak for yourself?**						
I have not thought about this	220 (22.1)	11 (9.8)	23 (15.3)	74 (22.4)	112 (27.7)	<.001
I have thought about this but have not talked to anyone about it	129 (13.0)	10 (8.9)	15 (10.0)	53 (16.1)	51 (12.6)	.03
I have talked about this with a friend or family member but have not signed official papers	252 (25.3)	21 (18.8)	33 (22.0)	103 (31.2)	95 (23.5)	.01
I have talked about this with a doctor or other health care provider but have not signed official papers	81 (8.1)	8 (7.1)	11 (7.3)	29 (8.8)	33 (8.2)	.16
I have signed official papers	376 (37.8)	67 (59.8)	75 (50.0)	97 (29.4)	137 (33.9)	<.001
Missing	6 (0.6)	0	0	0	6 (1.5)	.03
**If you had to decide right now, would you want CPR (cardiopulmonary resuscitation) if your heart were to stop beating?**						
Definitely yes	644 (64.7)	37 (33.0)	93 (62.0)	255 (77.3)	259 (64.1)	<.001
Probably yes	193 (19.4)	38 (33.9)	33 (22.0)	45 (13.6)	77 (19.1)	
Probably not	63 (6.3)	18 (16.1)	7 (4.7)	15 (4.6)	23 (5.7)	
Definitely not	87 (8.7)	18 (16.1)	16 (10.7)	14 (4.2)	39 (9.7)	
Missing	9 (0.9)	1 (0.9)	1 (0.7)	1 (0.3)	6 (1.5)	
**If you had to decide right now, would you want to be placed on a breathing machine (ventilator or respirator) if you became so sick that you could not breathe on your own?**						
Definitely yes	366 (36.8)	21 (18.8)	47 (31.3)	135 (40.9)	163 (40.4)	<.001
Probably yes	265 (26.6)	29 (25.9)	44 (29.3)	94 (28.5)	98 (24.3)	
Probably not	168 (16.9)	35 (31.3)	20 (13.3)	49 (14.9)	64 (15.8)	
Definitely not	185 (18.6)	26 (23.2)	36 (24.0)	51 (15.5)	72 (17.8)	
Missing	12 (1.2)	1 (0.9)	3 (2.0)	1 (0.3)	7 (1.7)	
**If you were to become very sick in the future and were unable to speak for yourself, would you prefer**						
Extending life, even if that means having more pain and discomfort	190 (19.1)	5 (4.5)	17 (11.3)	95 (28.8)	73 (18.1)	<.001
Relieving pain and discomfort as much as possible, even if that means not living as long	481 (48.3)	90 (80.4)	96 (64.0)	130 (39.4)	165 (40.8)	
I’m not sure which I would choose	319 (32.0)	17 (15.2)	37 (24.7)	103 (31.2)	162 (40.1)	
Missing	6 (0.6)	0	0	2 (0.6)	4 (1.0)	
**If you had to decide right now, where would you prefer to die if circumstances allowed you to choose?**						
In my own home	522 (52.4)	72 (64.3)	78 (52.0)	172 (52.1)	200 (49.5)	.02
In the home of a relative or friend	52 (5.2)	6 (5.4)	10 (6.7)	15 (4.6)	21 (5.2)	
In a hospital	252 (25.3)	19 (17.0)	43 (28.7)	83 (25.2)	107 (26.5)	
In a nursing home	21 (2.1)	6 (5.4)	4 (2.7)	2 (0.6)	9 (2.2)	
Other	130 (13.1)	9 (8.0)	14 (9.3)	51 (15.5)	56 (13.9)	
Missing	19 (1.9)	0	1 (0.7)	7 (2.1)	11 (2.7)	

Abbreviation: USTATE, United States Renal Data System Study of Treatment Preferences.

^a^ Columns may not sum to 100% owing to rounding. Documentation of treatment preferences does not sum to 100% because participants could choose more than 1 option.

^b^ *P* values were calculated using a χ^2^ test (values for documentation of treatment preferences were calculated for each response because participants could choose more than 1 option).

### Multivariate Analyses Among Survey Participants

In analyses adjusted for all self-reported participant characteristics, participants 75 years or older (adjusted odds ratio [aOR], 0.2; 95% CI, 0.1-0.3) and those with fair or poor self-reported health status (aOR, 0.2; 95% CI, 0.1-0.3) were less likely to have a prognostic expectation of more than 10 years (vs <5 years), whereas those who self-identified as black (aOR, 2.2; 95% CI, 1.1-4.1) and those who reported that their spiritual beliefs were definitely important (aOR, 1.7; 95% CI, 1.0-2.7) were more likely. Patients who self-identified as black (aOR, 2.6; 95% CI, 1.4-4.9) or other race (aOR, 2.0; 95% CI, 1.0-4.0) were more likely to be uncertain about prognosis (vs having a prognostic expectation <5 years), whereas those with a college or postgraduate education (aOR, 0.6; 95% CI, 0.4-1.0) and those with fair or poor self-reported health status (aOR, 0.4; 95% CI, 0.3-0.6) were less likely to be uncertain about their prognosis (eTable 2 in the Supplement).

**Figure 3.**
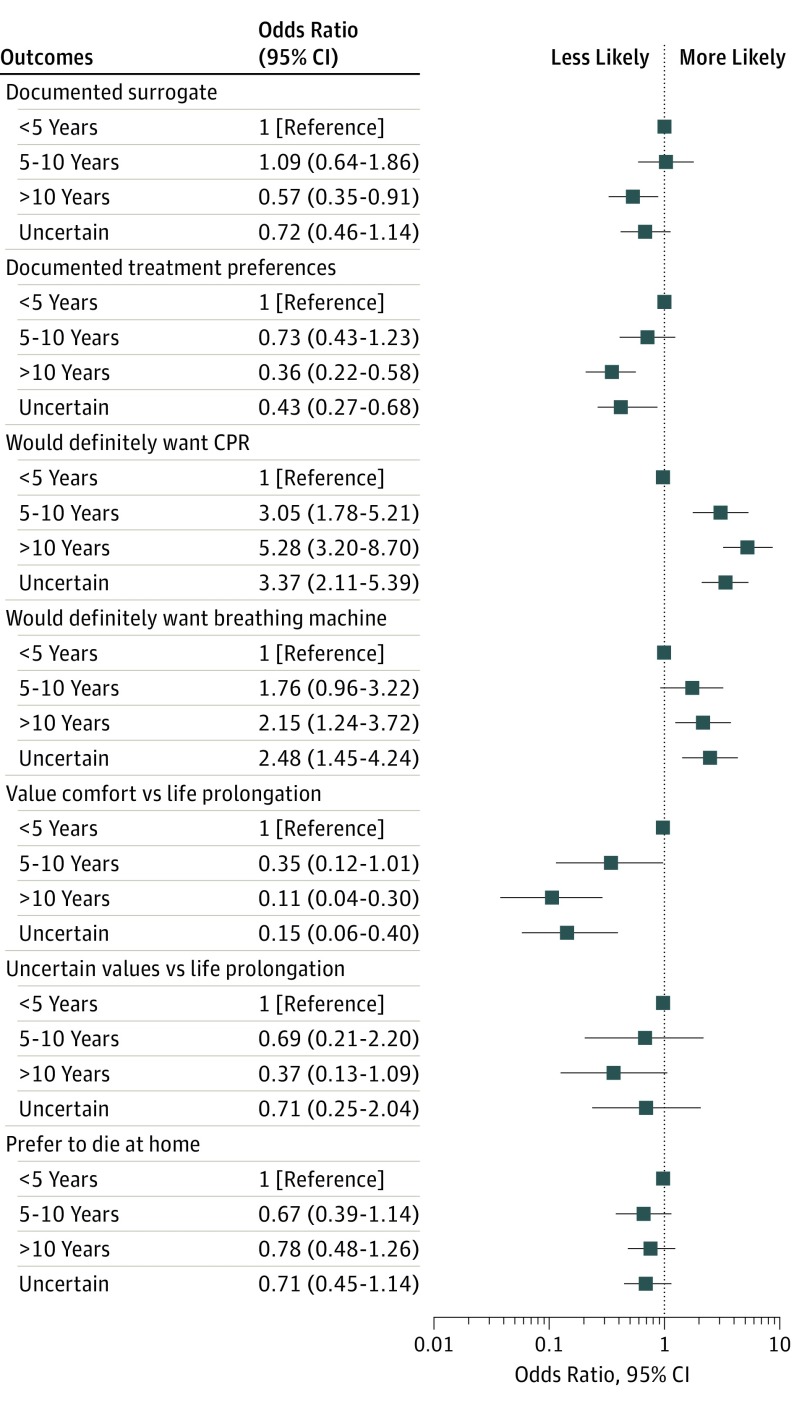
Adjusted Association of Prognostic Expectations With Engagement in Advance Care Planning, Values Around Life Prolongation, Treatment Preferences, and Preferred Place of Death For responses to the question on values, those valuing comfort and those who were uncertain were compared with the reference group of those valuing life prolongation. Analyses are adjusted for self-reported age, sex, race and ethnicity, health status, time undergoing dialysis, highest educational level, and spirituality. CPR indicates cardiopulmonary resuscitation.

After adjustment for all self-reported characteristics, participants with a prognostic expectation of more than 10 years (vs <5 years) were less likely to report documentation of a surrogate decision-maker (aOR, 0.6; 95% CI, 0.4-0.9) and treatment preferences (aOR, 0.4; 95% CI, 0.2-0.6) and to value comfort over life extension (aOR, 0.1; 95% CI, 0.04-0.3), and more likely to “definitely” want CPR (aOR, 5.3; 95% CI, 3.2-8.7) and mechanical ventilation (aOR, 2.2; 95% CI, 1.2-3.7) (Figure 3). Those who were uncertain about prognosis were less likely to have documented their treatment preferences (aOR, 0.4; 95% CI, 0.3-0.7) and to prefer a focus on comfort vs life prolongation (aOR, 0.2; 95% CI, 0.1-0.4), and were more likely to want CPR (aOR, 3.4; 95% CI, 2.1-5.4) and mechanical ventilation (aOR, 2.5; 95% CI, 1.5-4.2).

## Discussion

Consistent with the results of earlier studies of patients with advanced kidney disease,^21,24,26,27^ the prognostic expectations of participants in the present study were extremely optimistic. When asked to guess how long people of their age with similar health conditions usually live, 11.2% of survey participants responded fewer than 5 years, 15.1% responded 5 to 10 years, 33.1% responded more than 10 years, and 40.6% were not sure. These prognostic estimates contrast with the 60.3% 5-year mortality rate observed in the overall US in-center hemodialysis population.

Prognostic optimism and uncertainty in patients receiving dialysis likely reflect a number of different factors. Communication about prognosis is especially challenging for technologies such as dialysis, which promise to extend life and that shift the focus of care away from prognosis toward diagnosis and treatment.^8,30,31,32,33,34^ When dialysis is framed as a necessary lifesaving treatment rather than an explicit treatment choice, discussions about life expectancy, future course of illness, and treatment alternatives likely assume a lower priority.^35,36,37,38^ Although a number of tools have been developed to predict mortality in the dialysis population,^39,40^ estimating life expectancy in individual patients continues to be fraught with difficulty. Furthermore, payment and incentive structures for nephrologists and dialysis facilities have not traditionally encouraged—and may even discourage—frank discussions about prognosis.^34^ These conversations may be difficult, and nephrologists often do not feel equipped to have them.^23^

Little is known about the factors that shape the prognostic awareness of people undergoing dialysis. Prior work suggests that these patients’ prognostic expectations may be linked to expectations around kidney transplant.^24^ Although our survey did not include questions about transplant, it is striking that survey participants’ overall prognostic estimates most closely approximated actuarial survival for the small and relatively young segment of the US dialysis population that went on to receive a transplant, of whom more than 70% lived longer than 10 years. Spiritual and religious beliefs also seemed important in shaping prognostic expectations, which adds to previous work demonstrating the importance of spirituality in patients with advanced kidney disease.^41,42,43^ Collectively, these findings argue for more research to understand sources of prognostic knowledge among patients undergoing dialysis, more attention to communication around prognosis in nephrology training and continuing education programs, and stronger efforts to cultivate prognostic awareness and manage prognostic uncertainty among patients receiving dialysis.^33,42,43,44,45,46,47^

We found a strong association between expected prognosis and other domains of end-of-life care such that uncertainty about prognosis and more optimistic responses to the prognosis question were associated with decreased readiness to engage in advance care planning, as well as preferences for more aggressive treatment and prioritizing life extension over comfort. Collectively, these findings—which are consistent with prior work in patients undergoing dialysis, older adults, and people with cancer^1,2,3,24^—suggest that poor prognostic awareness and prognostic uncertainty may serve as barriers to shared decision-making and advance care planning, and perhaps contribute to intensive and costly patterns of end-of-life care in this population. The findings of this study also suggest that preparing patients undergoing dialysis for end-of-life decision-making might benefit from efforts to raise prognostic awareness and manage prognostic uncertainty.

### Limitations

This study has limitations. First, our question about prognosis has not been validated. We chose not to use existing instruments that ask patients to estimate their probability of surviving for prespecified periods of time because we felt that asking people to estimate life expectancy would better elicit their thinking about prognosis. We also wanted participants to reflect on the likely prognosis for someone like them to minimize the psychological defenses that might contribute to overly optimistic estimates. Although it is reassuring that none of the study participants reported difficulty understanding the prognosis question during pilot testing, it is possible that they may have interpreted this question in different ways. Second, because of differences in time period and in measured (eg, race and ethnicity), and perhaps unmeasured (eg, comorbid conditions), patient characteristics, it is possible that mortality rates among survey participants might differ from those reported for the USRDS cohort. We consider this unlikely because the survey had very few eligibility criteria, the response rate was relatively high, and the demographic composition of survey participants was similar to that of the prevalent US in-center hemodialysis population. Third, because we did not ask patients whether they had discussed prognosis with a clinician, or about their expectations for kidney transplant, our results provide limited insight into reasons for the high frequency of optimistic and uncertain prognostic expectations reported here. Fourth, results may not be generalizable to groups that were not included in the survey (eg, non-English–speaking patients, those who cannot provide informed consent, those dialyzing at for-profit facilities, and those living in rural or other US metropolitan areas). The generalizability of study results to patients receiving home hemodialysis or peritoneal dialysis may also be limited because nearly all participants were receiving in-center hemodialysis. Finally, though prognostic estimates were associated with treatment preferences and values, this does not imply a causal relationship. Further studies are needed to determine whether interventions to raise prognostic awareness can shape treatment preferences, values, quality of life, and preparedness for end-of-life care in this population.

## Conclusions

In summary, in our survey of patients receiving dialysis, most respondents were either uncertain about prognosis or had highly optimistic prognostic expectations when viewed in the context of actuarial survival in the US hemodialysis population. More optimistic and uncertain prognostic expectations among survey respondents were significantly associated with less engagement in advance care planning and preferences for life-extending treatment choices, which suggests that these expectations may limit the benefit of advance care planning and contribute to intensive and costly patterns of end-of-life care in this population. These findings highlight the need for a deeper understanding of what drives the prognostic expectations of patients undergoing dialysis and call for efforts to raise prognostic awareness and manage prognostic uncertainty among members of this population.

## Notes

Supplement. Supplemental Tables, Figure, and Survey. **eTable 1.** Characteristics of prevalent in-center hemodialysis patients **eTable 2.** Adjusted association of self-reported patient characteristics with prognostic expectations **eFigure.** Cohort derivation **eAppendix.** USRDS Study about Treatment Preferences (UState) Patient Questionnaire
