# Supplementary material for: Assessment of Self-reported Prognostic Expectations of People Undergoing Dialysis: United States Renal Data System Study of Treatment Preferences (USTATE)
Source: JAMA Intern Med. 2019 Jul 8;179(10):1325–33. doi: 10.1001/jamainternmed.2019.2879 (PMC6618699; doi:10.1001/jamainternmed.2019.2879)
Supplement: Supplement. — Supplemental Tables, Figure, and Survey. eTable 1. Characteristics of prevalent in-center hemodialysis patients eTable 2. Adjusted association of self-reported patient characteristics with prognostic expectations eFigure. Cohort derivation eAppendix. USRDS Study about Treatment Preferences (UState) Patient Questionnaire [file jamainternmed-179-1325-s001.pdf]

Supplementary Online Content

O'Hare AM, Kurella Tamura M, Lavallee DC, et al. Assessment of self-reported prognostic expectations of people on dialysis: United States Renal Data System Study of Treatment Preferences (USTATE). Published online July 8, 2019. *JAMA Intern Med*. doi:10.1001/jamainternmed.2019.2879

eTable 1. Characteristics of prevalent in-center hemodialysis patients
eTable 2. Adjusted association of self-reported patient characteristics with prognostic expectations
eFigure. Cohort derivation
eAppendix. USRDS Study about Treatment Preferences (UState) Patient Questionnaire

This supplementary material has been provided by the authors to give readers additional information about their work.

© 2019 O'Hare AM, et al. JAMA Intern Med.

eTable 1: Characteristics of prevalent in-center hemodialysis patients

	All patients (n=307,602)	Transplant recipients (n=33,713)
**Age group, %**		
<45 y	14.2	35.6
45-59 y	29.0	42.4
60-74 y	34.6	21.0
≥75 y	22.2	1.0
**Female gender, %**	45.3	36.7
**Race, %**		
White	55.1	51.7
Black	38.0	39.7
Asian	3.4	5.1
American Indian or Alaskan Native	1.5	1.5
Native Hawaiian or other Pacific Islander	1.1	1.2
Other or missing	0.9	0.9
**Ethnicity, %**		
Hispanic	15.7	21.0

© 2019 O'Hare AM, et al. JAMA Intern Med.

Missing	0.6	0.3
**Time since ESRD onset, %**		
<6 months	12.8	13.9
6-12 months	11.4	13.4
1-2 years	17.7	20.9
2-5 years	32.1	35.4
5-10 years	17.5	13.8
>10 years	8.5	2.6

Abbreviations: SD standard deviation; ESRD end-stage renal disease

© 2019 O'Hare AM, et al. JAMA Intern Med.

4 eTable 2: Adjusted association of self-reported patient characteristics with prognostic expectations

	**Prognostic expectations**			
	**Adjusted odds ratio (95% confidence interval)**			
	<5 years (n=112)	5-10 years (n=150)	>10 years (n=330)	Uncertain (n=404)
**Age ≥75 years**	1.0 (referent)	0.7 (0.4, 1.2)	0.2 (0.1, 0.3)	0.7 (0.4, 1.1)
**Female gender**	1.0 (referent)	0.8 (0.5, 1.3)	1.3 (0.8, 2.1)	0.8 (0.5, 1.2)
**Race**				
White	1.0 (referent)	1.0 (referent)	1.0 (referent)	1.0 (referent)
Black	1.0 (referent)	1.7 (0.85, 3.48)	2.2 (1.1, 4.1)	2.6 (1.4, 4.9)
Other	1.0 (referent)	0.8 (0.3, 1.8)	1.2 (0.6, 2.5)	2.0 (1.0, 4.0)
**Hispanic ethnicity**	1.0 (referent)	1.0 (0.3, 3.4)	2.2 (0.8, 6.0)	1.5 (0.5, 4.1)
**Fair or poor self-reported health status**	1.0 (referent)	0.5 (0.3, 0.8)	0.2 (0.1, 0.3)	0.4 (0.3, 0.6)
**> 2 years on dialysis**	1.0 (referent)	1.4 (0.8, 2.3)	1.0 (0.6, 1.5)	1.0 (0.7, 1.6)
**College or postgraduate education**	1.0 (referent)	1.3 (0.8, 2.2)	1.0 (0.6, 1.5)	0.6 (0.4, 1.0)
**Spiritual beliefs definitely important**	1.0 (referent)	2.0 (1.2, 3.3)	1.7 (1.0, 2.7)	1.4 (0.9, 2.2)

© 2019 O'Hare AM, et al. JAMA Intern Med.

Adjusted for self-reported age, sex, race, ethnicity, health status, time on dialysis, highest educational level and spirituality. For race we report the results of multinomial regression in which patients of white race with a prognostic expectation of <5 years serve as the referent category for all comparisons.

© 2019 O'Hare AM, et al. JAMA Intern Med.

eFigure: Cohort derivation

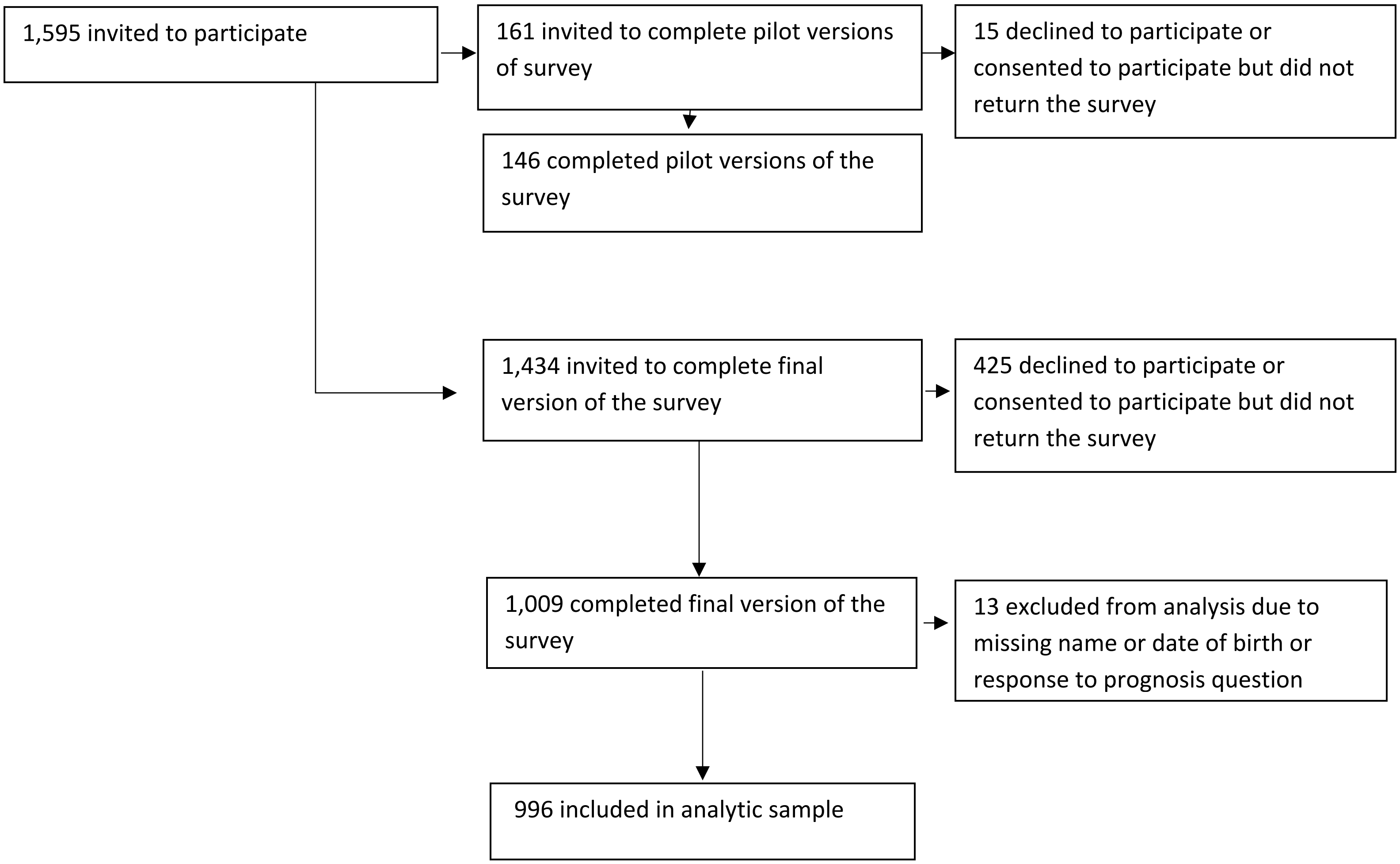

© 2019 O'Hare AM, et al. JAMA Intern Med.

USRDS Study about Treatment Preferences (UState) Patient Questionnaire

Thank you for taking part in our study to improve care for patients with kidney disease. We want to hear about the experiences of people receiving dialysis. We hope that by improving our understanding of the experiences of patients on dialysis and their families that we will be able to better help patients receive the type of care they need and want.

This questionnaire covers a number of topics. Some questions are about your health and emotions. Another set of questions are about the care preferences you may have now or if you become very sick. Other questions ask for general information about you, so that we can find out more about the people who are participating in this study. It will take about 30 minutes to complete these questions.

Because many types of people will be answering these questions, some questions may not apply to you. Please feel free to skip any questions that you do not want to answer, or that you feel do not apply to you. Because we have used questions from other surveys, some of the questions may seem quite similar to each other. All of your answers are confidential and will not be shared with anyone else.

Thank you very much for taking the time to complete this survey.

© 2019 O'Hare AM, et al. JAMA Intern Med.

Personal information

Your name: _______________ _______________ ____________________

First Middle Family name

Your date of birth: ____/_____/_____

Mo. / Day / Year

Your social security number: ______ ____ ______ *(only include if you agree that your social security number can be used for linkage to USRDS)*

Is someone helping you to answer these questions?

☐ Yes

☐ No

Today's date:

Today's date: ____________/ ______________/ ____________________

Month Day Year

© 2019 O'Hare AM, et al. JAMA Intern Med.

Section A: Your Overall Health and Symptoms

This section asks about your overall health and symptoms that you have had in the last week.

A1. In general, would you say your health is: ***(Check only one answer)***

☐ Excellent

☐ Very good

☐ Good

☐ Fair

☐ Poor

Questions A2-A19: Please check either the **"yes" or "no"** boxes to the right of each symptom listed below to report whether or not you have had that symptom **over the last week**

	**Symptom**	**Have you had this symptom in the last week?**	
		**Yes**	**No**
**A2.**	Pain		
**A3.**	Shortness of breath		
**A4.**	Weakness or lack of energy		
**A5.**	Nausea (feeling like you are going to vomit or throw up)		
**A6.**	Vomiting (throwing up)		

© 2019 O'Hare AM, et al. JAMA Intern Med.

	Symptom	Have you had this symptom in the last week?	
		Yes	No
**A7.**	Poor appetite		
**A8.**	Constipation		
**A9.**	Mouth problems		
**A10.**	Drowsiness		
**A11.**	Poor mobility		
**A12.**	Itching		
**A13.**	Difficulty sleeping		
**A14.**	Restless legs or difficulty keeping legs still		
**A15.**	Feeling anxious		
**A16.**	Feeling depressed		
**A17.**	Changes in your skin		
**A18.**	Diarrhea		
**A19.**	Which symptom has bothered you the most over the past week? Describe:		

Please go on to the next page-------------------------------->

© 2019 O'Hare AM, et al. JAMA Intern Med.

Section B: Your Needs

This section asks about your spiritual, educational and care needs.

Questions B1-B18: Please check either the **"yes" or "no"** box to the right of each question below. Although we will not be able to meet your needs as part of this survey, your answers to these questions are helpful for our research.

**I would like to learn more about:**		**Yes**	**No**
**B1.**	How to be in touch with other patients with kidney disease		
**B2.**	What I can do about pain		
**B3.**	Relaxation or stress management		
**B4.**	Treating the symptoms of kidney disease (itching, nausea, fatigue)		

**I would like help with:**		**Yes**	**No**
**B5.**	Making plans in case I become very ill		
**B6.**	Learning to cope with feelings of sadness		
**B7.**	Sharing my thoughts and feelings with those close to me		
**B8.**	Finding spiritual resources		

© 2019 O'Hare AM, et al. JAMA Intern Med.

I would like help with:		Yes	No
**B9.**	Worries I have about the effect of my illness on my family		
**B10.**	Finding meaning in my life now		
**B11.**	Finding hope		
**B12.**	Overcoming my fears		
**B13.**	Organizing my appointments and treatments		
**III. I would like to have someone to talk to about:**		**Yes**	**No**
**B14.**	Talking about my care plan and treatments		
**B15.**	Treatment options for the future		
**B16.**	The meaning of life		
**B17.**	Dying and death		
**B18.**	Finding peace of mind		

© 2019 O'Hare AM, et al. JAMA Intern Med.

Section C: Planning for Serious Illness

This section asks about planning for your future healthcare if you were to become very sick in the future.

C1. Do you have a person who could make medical decisions for you if you were to become very sick and were unable to speak for yourself? *(This is known as a surrogate decision-maker, durable power of attorney, or DPOA)* ***(Check only one answer)***

☐ I have not thought about this

☐ I have thought about this, but *have not decided who* this would be

☐ I know who this would be, but *have not asked* him/her

☐ I have asked someone, but *have not signed official papers* naming him/her as the person who will make medical decisions for me

☐ I have signed official papers naming someone to make medical decisions for me (e.g., as part of a living will or advance directive), but *have not discussed* this with him/her

☐ I have signed official papers naming someone to make medical decisions for me (e.g., as part of a living will or advance directive), and *have discussed* this with him/her

© 2019 O'Hare AM, et al. JAMA Intern Med.

C2. Have you thought about the kinds of treatments that you would want or not want if you were to become very sick and were unable to speak for yourself? **(*Check all answers that apply)***

☐ I have not thought about this

☐ I have thought about this, but *have not talked to anyone* about it

☐ I have talked about this with a friend or family member, but *have not signed official papers*

☐ I have talked about this with a doctor or other healthcare provider, but *have not signed official papers*

☐ I have signed official papers documenting my preferences (e.g., living will or advance directive), but *have not talked with any friends or family members* about this

☐ I have signed official papers documenting my preferences (e.g., living will or advance directive), *and have talked with at least one friend or family member* about this

© 2019 O'Hare AM, et al. JAMA Intern Med.

C3. If you were to become very sick in the future and were unable to speak for yourself, would you prefer a plan of medical care that focuses on extending life as much as possible, even if it means having more pain and discomfort, or would you want a plan of medical care that focuses on relieving pain and discomfort as much as possible, even if that means not living as long? ***(Check only one answer)***

☐ Extending life, even if that means having more pain and discomfort

☐ Relieving pain and discomfort as much as possible, even if that means not living as long

☐ I'm not sure which I would choose

C4. If you had to decide right now, would you want CPR (cardiopulmonary resuscitation) if your heart were to stop beating? ***(Check only one answer)***

☐ Definitely yes

☐ Probably yes

☐ Probably not

☐ Definitely not

© 2019 O'Hare AM, et al. JAMA Intern Med.

C5. If you had to decide right now, would you want to be placed on a breathing machine (ventilator or respirator) if you became so sick that you could not breathe on your own? ***(Check only one answer)***

☐ Definitely yes

☐ Probably yes

☐ Probably not

☐ Definitely not

C6. If you had to decide right now, where would you prefer to die if circumstances allowed you to choose? ***(Check only one answer)***

☐ In my own home

☐ In the home of a relative or friend

☐ In a hospital

☐ In a nursing home

☐ Other: ________________________________ (describe)

© 2019 O'Hare AM, et al. JAMA Intern Med.

C7. If you were to become very sick in the future and were facing a decision about whether to accept treatments to prolong your life that might increase your suffering, <u>*what role would you want to have*</u> in that decision? ***(Check only one answer)***

☐ I prefer to make the final selection about which treatments I will receive

☐ I prefer to make the final selection of my treatment after seriously considering my doctor's opinion.

☐ I prefer that my doctor and I share responsibility for deciding which treatments are best for me.

☐ I prefer that my doctor makes the final decision about which treatments will be used, but seriously considers my opinion.

☐ I prefer to leave all treatment decisions to my doctor.

© 2019 O'Hare AM, et al. JAMA Intern Med.

C8. Have you *ever thought about* stopping your dialysis treatments? ***(Check only one answer)***

☐ Yes

☐ No

C9. Have you *ever had a discussion* about the option of stopping dialysis if you were to become sicker, or if your goals changed? ***(Check all answers that apply)***

☐ Yes, with my kidney doctor

☐ Yes, with my primary care doctor

☐ Yes, with a nurse

☐ Yes, with a social worker

☐ Yes, with another healthcare provider: _______________(describe)

☐ Yes, with a friend or family member

☐ No, I have never had a discussion about this with anyone

© 2019 O'Hare AM, et al. JAMA Intern Med.

C10. Have you *ever thought about* whether you might want to receive hospice care if you were to become sicker or if your goals changed? (This is care that is focused on trying to keep people comfortable toward the end of life rather than trying to prolong life.) ***(Check only one answer)***

☐ Yes

☐ No

C11. Have you *ever had a discussion* about the option of receiving hospice care if you were to become sicker or if your goals changed? (This is care that is focused on trying to keep people comfortable toward the end of life rather than trying to prolong life.) ***(Check all answers that apply)***

☐ Yes, with my kidney doctor

☐ Yes, with my primary care doctor

☐ Yes, with a nurse

☐ Yes, with a social worker

☐ Yes, with another healthcare provider: ________________(describe)

☐ Yes, with a friend or family member

☐ No, I have never had a discussion about this with anyone

© 2019 O'Hare AM, et al. JAMA Intern Med.

C12. How long would you guess people your age with similar health conditions usually live? ***(Check only one answer)***

☐ Less than 6 months

☐ 6 to 12 months

☐ 1 to 2 years

☐ 2 to 5 years

☐ 5 to 10 years

☐ More than 10 years

☐ I'm not sure

© 2019 O'Hare AM, et al. JAMA Intern Med.

Section D: About You

The next questions provide us with information about you so that we will be able to describe the people who completed this questionnaire.

D1. What is your gender?

☐ Female

☐ Male

☐ Other: ________________________________ (describe)

D2. What ethnicity do you consider yourself? ***(Check only one answer)***

☐ Non-Hispanic

☐ Hispanic

D3. What race do you consider yourself? ***(Check only one answer)***

☐ White

☐ Black or African American

☐ Asian

☐ American Indian or Alaskan native

☐ Native Hawaiian or other Pacific Islander

☐ Other: ________________________________ (describe)

© 2019 O'Hare AM, et al. JAMA Intern Med.

D4. What is the highest level of education you have completed? ***(Check only one answer)***

☐ 8th grade or less

☐ Some high school

☐ Graduated from high school

☐ Graduated from college, community college or trade school

☐ Other: ________________________________ (describe)

D5. How true is the following statement for you? "My religious or spiritual beliefs are what really lie behind my whole approach to life." ***(Check only one answer)***

☐ Definitely true

☐ Tends to be true

☐ Tends not to be true

☐ Definitely not true

© 2019 O'Hare AM, et al. JAMA Intern Med.

D6. Which religion/spiritual group do you belong to? ***(Check all answers that apply)***

☐ Christian

☐ Buddhist

☐ Muslim

☐ Jewish

☐ None

☐ Other: ________________________________ (describe)

D7. What type of dialysis treatment are you currently receiving? ***(Check only one answer)***

☐ Hemodialysis

☐ Peritoneal dialysis

© 2019 O'Hare AM, et al. JAMA Intern Med.

D8. How long have you been on dialysis? ***(Check only one answer)*** *If you can't decide which of two categories your answer fits into, please choose the higher of the two categories.*

☐ Less than 6 months

☐ 6 to 12 months

☐ 1 to 2 years

☐ 2 to 5 years

☐ 5 to 10 years

☐ More than 10 years

☐Other: ________________________________ (describe)

© 2019 O'Hare AM, et al. JAMA Intern Med.

Section E. Contact Information for Family Members

As part of this study, we are hoping to contact one or more of your family members or friends to invite them to participate in this study.

☐ I do not have anyone to ask

☐ I don't want the study team to contact my family members or friends

If you are willing for the study team to contact close friends and/or family members, please list contact information for one or more adult family members or friends to invite to participate in this study below. Please list the family member or friend who is most involved in your care first.

Name of friend or family member: ______________________________

Mailing Address: ______________________________

____

____

Best telephone # (with area code): ______________________________

Email address: ______________________________

Relationship to you: ______________________________

Name of friend or family member: ______________________________

Mailing Address: ______________________________

____

____

Best telephone # (with area code): ______________________________

Email address: ______________________________

Relationship to you: _____________________________

© 2019 O'Hare AM, et al. JAMA Intern Med.

Section F: Comments?

Do you have any thoughts or opinions about planning for future care that are important to you that we may have missed? Please feel free to tell us in your own words in the space provided below.

____

____

We would like to obtain your feedback on the questionnaire so that we can improve it. Your answers to the following questions will help us do this.

Were there any questions that were difficult to understand? *If so, please list the question number(s):*

____

Were there any questions that were upsetting? *If so, please list the question number(s)*

____

Can you explain to us what upset you about this/these question(s)?

____

____

Are there any questions you think we should have asked that were not included in the questionnaire? *If so, please tell us what these would have been.*

____

____

This is the end of this questionnaire. Thank you for taking the time to complete this questionnaire. If you have any questions, feel free to call us at: 206.616.8574

Thank you again for your help!

© 2019 O'Hare AM, et al. JAMA Intern Med.
